# Supplementary material for: Unveiling the Bioactive Potential of the Invasive Jellyfish Phyllorhiza punctata Through Integrative Transcriptomic and Proteomic Analyses
Source: Biomolecules. 2025 Aug 4;15(8):1121. doi: 10.3390/biom15081121 (PMC12383608; doi:10.3390/biom15081121)
Supplement: Supplementary file 1 [file biomolecules-15-01121-s001.zip › Suplementary Material/FileS3/PEPFOLD4/XP_065069084_10962/clusters.html]

 PEP-FOLD clusters


Download archive of all models  here   

|  |  |  |
| --- | --- | --- |
| # | file | sOPEP |
| # cluster 1 - Size: 10 |  |  |
|  | XP\_065069084\_10962-model1 | -12.6008 |
|  | XP\_065069084\_10962-model1.1 | -12.5809 |
|  | XP\_065069084\_10962-model1.2 | -12.5121 |
|  | XP\_065069084\_10962-model1.3 | -12.4739 |
|  | XP\_065069084\_10962-model1.4 | -12.4695 |
|  | XP\_065069084\_10962-model1.5 | -12.4341 |
|  | XP\_065069084\_10962-model1.6 | -12.4044 |
|  | XP\_065069084\_10962-model1.7 | -12.3796 |
|  | XP\_065069084\_10962-model1.8 | -12.2003 |
|  | XP\_065069084\_10962-model1.9 | -12.0525 |
| # cluster 2 - Size: 3 |  |  |
|  | XP\_065069084\_10962-model2 | -8.0056 |
|  | XP\_065069084\_10962-model2.1 | -7.0925 |
|  | XP\_065069084\_10962-model2.2 | -6.84965 |
| # cluster 3 - Size: 10 |  |  |
|  | XP\_065069084\_10962-model3 | -7.33661 |
|  | XP\_065069084\_10962-model3.1 | -7.00887 |
|  | XP\_065069084\_10962-model3.2 | -6.95436 |
|  | XP\_065069084\_10962-model3.3 | -6.93978 |
|  | XP\_065069084\_10962-model3.4 | -6.83012 |
|  | XP\_065069084\_10962-model3.5 | -6.82016 |
|  | XP\_065069084\_10962-model3.6 | -6.76255 |
|  | XP\_065069084\_10962-model3.7 | -6.68989 |
|  | XP\_065069084\_10962-model3.8 | -6.65852 |
|  | XP\_065069084\_10962-model3.9 | -6.65727 |
| # cluster 4 - Size: 14 |  |  |
|  | XP\_065069084\_10962-model4 | -7.32573 |
|  | XP\_065069084\_10962-model4.1 | -7.2902 |
|  | XP\_065069084\_10962-model4.2 | -7.19366 |
|  | XP\_065069084\_10962-model4.3 | -7.11509 |
|  | XP\_065069084\_10962-model4.4 | -7.11352 |
|  | XP\_065069084\_10962-model4.5 | -7.06755 |
|  | XP\_065069084\_10962-model4.6 | -7.04354 |
|  | XP\_065069084\_10962-model4.7 | -6.99861 |
|  | XP\_065069084\_10962-model4.8 | -6.98573 |
|  | XP\_065069084\_10962-model4.9 | -6.9641 |
|  | XP\_065069084\_10962-model4.10 | -6.87506 |
|  | XP\_065069084\_10962-model4.11 | -6.86022 |
|  | XP\_065069084\_10962-model4.12 | -6.72107 |
|  | XP\_065069084\_10962-model4.13 | -6.721 |
| # cluster 5 - Size: 8 |  |  |
|  | XP\_065069084\_10962-model5 | -7.22843 |
|  | XP\_065069084\_10962-model5.1 | -7.05324 |
|  | XP\_065069084\_10962-model5.2 | -7.05324 |
|  | XP\_065069084\_10962-model5.3 | -6.99923 |
|  | XP\_065069084\_10962-model5.4 | -6.99923 |
|  | XP\_065069084\_10962-model5.5 | -6.99783 |
|  | XP\_065069084\_10962-model5.6 | -6.99377 |
|  | XP\_065069084\_10962-model5.7 | -6.89569 |
| # cluster 6 - Size: 10 |  |  |
|  | XP\_065069084\_10962-model6 | -7.22256 |
|  | XP\_065069084\_10962-model6.1 | -7.1346 |
|  | XP\_065069084\_10962-model6.2 | -7.12835 |
|  | XP\_065069084\_10962-model6.3 | -7.12028 |
|  | XP\_065069084\_10962-model6.4 | -7.11805 |
|  | XP\_065069084\_10962-model6.5 | -7.11805 |
|  | XP\_065069084\_10962-model6.6 | -7.10311 |
|  | XP\_065069084\_10962-model6.7 | -6.96661 |
|  | XP\_065069084\_10962-model6.8 | -6.94487 |
|  | XP\_065069084\_10962-model6.9 | -6.8614 |
| # cluster 7 - Size: 19 |  |  |
|  | XP\_065069084\_10962-model7 | -7.06658 |
|  | XP\_065069084\_10962-model7.1 | -7.03102 |
|  | XP\_065069084\_10962-model7.2 | -6.93537 |
|  | XP\_065069084\_10962-model7.3 | -6.90665 |
|  | XP\_065069084\_10962-model7.4 | -6.90665 |
|  | XP\_065069084\_10962-model7.5 | -6.86772 |
|  | XP\_065069084\_10962-model7.6 | -6.86221 |
|  | XP\_065069084\_10962-model7.7 | -6.86221 |
|  | XP\_065069084\_10962-model7.8 | -6.82712 |
|  | XP\_065069084\_10962-model7.9 | -6.82401 |
|  | XP\_065069084\_10962-model7.10 | -6.81883 |
|  | XP\_065069084\_10962-model7.11 | -6.81088 |
|  | XP\_065069084\_10962-model7.12 | -6.80224 |
|  | XP\_065069084\_10962-model7.13 | -6.79565 |
|  | XP\_065069084\_10962-model7.14 | -6.76728 |
|  | XP\_065069084\_10962-model7.15 | -6.75888 |
|  | XP\_065069084\_10962-model7.16 | -6.75859 |
|  | XP\_065069084\_10962-model7.17 | -6.75726 |
|  | XP\_065069084\_10962-model7.18 | -6.59935 |
| # cluster 8 - Size: 18 |  |  |
|  | XP\_065069084\_10962-model8 | -6.95938 |
|  | XP\_065069084\_10962-model8.1 | -6.81596 |
|  | XP\_065069084\_10962-model8.2 | -6.80956 |
|  | XP\_065069084\_10962-model8.3 | -6.80201 |
|  | XP\_065069084\_10962-model8.4 | -6.80201 |
|  | XP\_065069084\_10962-model8.5 | -6.78902 |
|  | XP\_065069084\_10962-model8.6 | -6.78503 |
|  | XP\_065069084\_10962-model8.7 | -6.77068 |
|  | XP\_065069084\_10962-model8.8 | -6.76286 |
|  | XP\_065069084\_10962-model8.9 | -6.74498 |
|  | XP\_065069084\_10962-model8.10 | -6.74102 |
|  | XP\_065069084\_10962-model8.11 | -6.73693 |
|  | XP\_065069084\_10962-model8.12 | -6.71159 |
|  | XP\_065069084\_10962-model8.13 | -6.68646 |
|  | XP\_065069084\_10962-model8.14 | -6.68505 |
|  | XP\_065069084\_10962-model8.15 | -6.64425 |
|  | XP\_065069084\_10962-model8.16 | -6.63401 |
|  | XP\_065069084\_10962-model8.17 | -6.51807 |
| # cluster 9 - Size: 1 |  |  |
|  | XP\_065069084\_10962-model9 | -6.8895 |
| # cluster 10 - Size: 6 |  |  |
|  | XP\_065069084\_10962-model10 | -6.87769 |
|  | XP\_065069084\_10962-model10.1 | -6.83662 |
|  | XP\_065069084\_10962-model10.2 | -6.78987 |
|  | XP\_065069084\_10962-model10.3 | -6.78763 |
|  | XP\_065069084\_10962-model10.4 | -6.77977 |
|  | XP\_065069084\_10962-model10.5 | -6.70411 |
| # cluster 11 - Size: 1 |  |  |
|  | XP\_065069084\_10962-model11 | -6.64785 |
|  |  |  |
